# Training satisfaction for subspecialty fellows in internal medicine: Findings from the Veterans Affairs (VA) Learners' Perceptions Survey

**DOI:** 10.1186/1472-6920-11-21

**Published:** 2011-05-17

**Authors:** Catherine P Kaminetzky, Sheri A Keitz, T Michael Kashner, David C Aron, John M Byrne, Barbara K Chang, Christopher Clarke, Stuart C Gilman, Gloria J Holland, Annie Wicker, Grant W Cannon

**Affiliations:** 1 Department of Medicine, Duke University, Durham NC, USA; 2 Department of Education, Durham VA Medical Center, Durham NC, USA; 3 Department of Medicine, Miami VA Health Care System, Miami FL, USA; 4 Department of Medicine, University of Miami Miller School of Medicine, Miami FL, USA; 5 Office of Academic Affiliations, Veterans Health Administration, Washington DC, USA; 6 Department of Medicine, Loma Linda University School of Medicine, Loma Linda CA, USA; 7 Education Office, Louis Stokes Cleveland Department of Veterans Affairs Medical Center, Cleveland OH, USA; 8 Department of Medicine, Case Western Reserve University School of Medicine, Cleveland, OH, USA; 9 Department of Education, Loma Linda VA Medical Center, Loma Linda CA, USA; 10 Center for Advanced Statistics in Education, Loma Linda VA Health Care System, Loma Linda CA, USA; 11 Department of Education, George E. Wahlen VA Medical Center, Salt Lake City, UT, USA; 12 Division of Rheumatology, University of Utah School of Medicine, Salt Lake City UT, USA

## Abstract

**Background:**

Learner satisfaction assessment is critical in the design and improvement of training programs. However, little is known about what influences satisfaction and whether trainee specialty is correlated. A national comparison of satisfaction among internal medicine subspecialty fellows in the Department of Veterans Affairs (VA) provides a unique opportunity to examine educational factors associated with learner satisfaction. We compared satisfaction across internal medicine fellows by subspecialty and compared factors associated with satisfaction between procedural versus non-procedural subspecialty fellows, using data from the Learners' Perceptions Survey (LPS), a validated survey tool.

**Methods:**

We surveyed 2,221 internal medicine subspecialty fellows rotating through VA between 2001 and 2008. Learners rated their overall training satisfaction on a 100-point scale, and on a five-point Likert scale ranked satisfaction with items within six educational domains: learning, clinical, working and physical environments; personal experience; and clinical faculty/preceptor.

**Results:**

Procedural and non-procedural fellows reported similar overall satisfaction scores (81.2 and 81.6). Non-procedural fellows reported higher satisfaction with 79 of 81 items within the 6 domains and with the domain of physical environment (4.06 vs. 3.85, p <0.001). Satisfaction with clinical faculty/preceptor and personal experience had the strongest impact on overall satisfaction for both. Procedural fellows reported lower satisfaction with physical environment.

**Conclusions:**

Internal medicine fellows are highly satisfied with their VA training. Nonprocedural fellows reported higher satisfaction with most items. For both procedural and non-procedural fellows, clinical faculty/preceptor and personal experience have the strongest impact on overall satisfaction.

## Background

The quality of training provided in physician training programs is an important focus of health systems, hospitals and undergraduate and graduate medical education leaders. Trainee satisfaction is one element of quality in clinical education. Its relationship with different components of clinical, learning and work experiences is often explored to identify elements associated with high satisfaction within individual training programs, but not across training programs nationally or across disciplines. Understanding which factors contribute to trainee satisfaction, and how they contribute, is critical to the design of education programs that will meet the needs of trainees across different specialties.

An ideal setting for examining components of trainee satisfaction is the Department of Veterans Affairs (VA). VA is the second largest funding source of United States (US) physician training positions, with over 9500 physician resident positions of which 1600 internal medicine subspecialty fellow positions were funded in 2008-2009. Each year, nearly one-third of the nation's physician trainees rotate at 120 VA centers and three independent outpatient clinics through affiliations with medical schools and teaching hospitals.

Previous studies of satisfaction with VA training have used data from the Learners' Perceptions Survey (LPS), a validated survey instrument that measures satisfaction across multiple domains. Since 2001, VA has administered the LPS annually to all learners who train at VA medical facilities. Prior work using data from the LPS established differences in perception of satisfaction for learners in different stages of training [1] and between physician trainees in different specialties [2]. One hypothesis to explain differences between types of trainees is that such variability may be related to differences in daily experiences. To test this hypothesis, we examined the degree to which predominance of procedural experiences explain dissimilarities among learners, focusing on fellows in different subspecialties in internal medicine.

Specifically, we measured satisfaction across internal medicine fellows by subspecialty and compared satisfaction between procedural versus non-procedural subspecialties. We also identified factors associated with satisfaction and compared how these factors differ between fellows in these two groups.

## Methods

### Survey development

The LPS was developed to examine and measure learner satisfaction for all healthcare trainees working in VA. Survey development began in 1999 using standard psychometric procedures [2]. An initial item pool was derived based on an exhaustive review of the literature on learner satisfaction and refined based on feedback from 15 focus groups of VA faculty and clinical trainees. Items were grouped into six domains: clinical faculty or preceptor (13 items), learning environment (15), clinical environment (15), working environment (13), physical environment (12) and personal experience (13). For each item, respondents were asked to rate satisfaction with VA training using a five-point Likert scale. The survey was piloted on over 1,000 trainees from 22 geographically diverse VA medical centers. Confirmatory factor analysis upheld the integrity of each domain. On the basis of pilot testing, the survey was refined to items that contributed to an overall training satisfaction and to satisfaction ratings in six educational domains. Items and the corresponding domains are listed in Additional file 1.

Since its 2001 rollout, the LPS has been administered annually to assess perceptions of clinical learners toward their VA experiences. The LPS consistently shows domain content stability, integrity and Alpha reliability in the .90s for both the overall survey and its sub-domains [2].

Over time, questions have been added to the LPS to explore the impact of changes in the clinical education environment on trainee satisfaction. For the current analysis, we evaluated only those components of the survey that have been unchanged since 2001, including scores for overall training satisfaction, educational domains and their associated items.

### LPS Outcome Measures

To derive an overall satisfaction score, learners are asked: "On a scale of 0 to 100, where 100 is a perfect score and 70 is a passing score, what numerical score would you give your most recent VA clinical training experience?" The response to this question is the "overall training satisfaction score" and the primary outcome measure used in this analysis.

### Study design

The current report presents survey results from an eight-year summary analysis of trainees' satisfaction with training experiences at VA medical centers from 2001 through 2008.

### Study population and survey administration

Participants were physician fellows in internal medicine fellowships who rotated through a VA facility during the academic year. In 2001, trainees registered for the LPS survey through a post-card registration process. Registered trainees were then mailed a paper survey or could complete an online version of the survey. For subsequent years the separate registration process was discontinued, and all physician trainees were encouraged to participate in the survey through a combination of national and local recruitment efforts. Nationally, letters of information and invitation were sent out from the Office of Academic Affiliations to all physician trainees for whom addresses were available. In addition, individual VA facilities were encouraged to develop complementary local processes to encourage trainee participation in the survey. Local processes for trainee recruitment varied. The survey was available in both paper and online versions in 2001 through 2003. Since 2004, the survey has been administered exclusively online.

We categorized fellows in internal medicine-based programs as participating in procedural fellowship (cardiology, gastroenterology and pulmonary/critical care medicine) or non-procedural fellowships (endocrinology, geriatrics, hematology/oncology, infectious diseases, nephrology and rheumatology).

### Analyses

We used mixed-effects models to adjust scores by subspecialty and to compute both the effect of individual items on domain scores and the effect of domain scores on overall satisfaction for the VA hospital. We adjusted all estimates to account for each subspecialty, calculated to reflect a PGY-4 fellow, and corrected for year of survey and facility nesting. Adjustment was necessary to permit comparisons across subspecialties when responders may be distributed across survey year and facilities differently. No adjustments were made to reflect characteristics of individual responders because the information was limited due to individual responder anonymity. Furthermore, we wanted to measure total, rather than partial, differences across specialties. We computed item effect sizes from the estimated coefficients to mean-centered domain item × subspecialty indicator interaction terms. Domain and domain items were scaled between one and five, where five indicates very satisfied, and one indicates very dissatisfied. We measured effect sizes (item on domain scores) to reflect the increase in domain score per unit increase in item score. Effect sizes were adjusted to reflect a PGY-4 and corrected for year of survey and facility nesting. We did not adjust to reflect changes in the other items since our purpose was to compute a total effect size. All procedures were performed using SPSS. To account for multiple comparisons, we consider statistically significant only factors where p ≤ 0.001.

### Ethical considerations

The U.S. Office of Management and Budget, which reviews and approves federal government sponsored surveys, approved the LPS. We maintained confidentiality by keeping respondent information in a separate database and reviewing only aggregate data. Participation in the survey was voluntary. The confidential nature of the data collection and voluntary participation were fully disclosed to survey participants.

## Results

### Overall training satisfaction

There were 2,221 responses by fellows between 2001 and 2008 (Table 1). The distribution of respondents by fellowship was very similar to the distribution of funded positions by the OAA for each fellowship type (data not shown). There were 1,026 responses in the procedural group and 1,195 responses in the non-procedural group. Cardiology fellows provided the largest number of responses for procedural fellows (n = 459), and Hematology-Oncology fellows provided the largest number of responses for non-procedural fellows (n = 283).

**Table 1:** Number of responders by year of survey, level of training and discipline

Responses	No. (% of Total)
Year of Survey	
2001	141 (6.4%)

2002	184 (8.3%)

2003	309 (13.9%)

2004	260 (11.7%)

2005	264 (11.9%)

2006	368 (16.6%)

2007	268 (12.1%)

2008	427 (19.2%)

*Total*	2221 (100%)

	

Post graduate year	

PGY-4	976 (43.9%)

PGY-5	671 (30.2%)

PGY-6	440 (19.8%)

PGY-7 and above	134 (6.0%)

*Total*	2221 (100%)

	

Procedural group	

Cardiology	459 (20.7%)

Gastroenterology	269 (12.1%)

Pulmonary	298 (13.4%)

Subtotal	1026 (46.2%)

	

Non-procedural group	

Endocrinology	155 (7.0%)

Geriatrics	264 (11.9%)

Hematology/Oncology	283 (12.7%)

Infectious Diseases	193 (8.7%)

Nephrology	191 (8.6%)

Rheumatology	109 (4.9%)

Subtotal	1195 (53.8%)

	

Total	2221 (100%)

Overall satisfaction scores were similar for all internal medicine subspecialties, revealing only minor differences in adjusted mean scores that did not achieve statistical significance (Table 2). Additionally, the mean adjusted overall satisfaction scores for procedural fellows (81.2) and non-procedural fellows (81.6) were not statistically significantly different (p = 0.59).

**Table 2:** Adjusted overall training satisfaction scores for resident fellows by subspecialty

Discipline	Adjusted overall score*
	(95% CI)
Procedural	
Cardiology	80.8 (79.3, 82.4)
Gastroenterology	83.6 (81.8, 85.4)
Pulmonary	79.5 (77.7, 81.2)
Subtotal	81.2 (79.0, 83.4)
	
Non-procedural	
Endocrinology	80.2 (78.0, 82.4)
Geriatric	83.8 (82.1, 85.4)
Hematology/Oncology	79.8 (78.1, 81.5)
Infectious Diseases	81.9 (79.8, 83.9)
Nephrology	80.5 (78.6, 82.5)
Rheumatology	84.2 (81.6, 86.6)
Subtotal	81.6 (80.6, 82.5)

* Scores range from 1 to 100, where 100 indicates very satisfied with overall training at VA, and are adjusted to reflect mean by subspecialty, calculated for PGY-4, and corrected for year of survey and facility nesting.

### Satisfaction at the domain level

Procedural and non-procedural fellows reported similar satisfaction with the following domains: clinical faculty/preceptors, personal experiences, learning, working and clinical environments. There were differences in the reported satisfaction with overall physical environment, with non-procedural fellows reporting higher satisfaction compared to procedural fellows (4.06 vs. 3.85, p < 0.001) (Table 3). Rank order of domain satisfaction was similar for procedural and non-procedural fellows, with trainees reporting highest satisfaction with clinical faculty/preceptors, followed by personal experience, learning environment and working environment.

**Table 3:** Adjusted mean scores for domains and selected* items for non-procedural and procedural fellows

	Non-procedural fellows	Procedural fellows		
Category	Mean (95% CI)**	Mean (95% CI)	*t*	*p*
Clinical faculty/preceptors	4.42 (4.36, 4.48)	4.32 (4.25, 4.40)	2.41	0.016
Accessibility/availability	4.48 (4.43, 4.54)	4.35 (4.28, 4.42)	3.35	0.001
Timeliness of feedback	4.33 (4.27, 4.40)	4.19 (4.11, 4.27)	3.30	0.001
Fairness in evaluation	4.46 (4.40, 4.52)	4.31 (4.24, 4.38)	3.66	<0.001
Patient-oriented	4.50 (4.44, 4.56)	4.37 (4.30, 4.44)	3.29	0.001
				
Personal experience	4.35 (4.29, 4.41)	4.24 (4.17, 4.31)	2.83	0.005
Continuity of relationship with patients	4.25 (4.17, 4.33)	4.07 (3.98, 4.17)	3.38	0.001
				
Learning environment	4.17 (4.10, 4.24)	4.11 (4.03, 4.19)	1.35	0.18
Time for learning	4.19 (4.11, 4.26)	3.99 (3.90, 4.08)	4.05	<0.001
Teaching conferences	4.02 (3.94, 4.10)	3.84 (3.75, 3.94)	3.23	0.001
				
Working environment	4.12 (4.04, 4.19)	4.00 (3.91, 4.08)	2.40	0.017
Ancillary/support staff morale	3.84 (3.74, 3.94)	3.54 (3.42, 3.66)	4.59	<0.001
Laboratory services	3.99 (3.91, 4.07)	3.81 (3.71, 3.91)	3.38	0.001
Ancillary/support staff	3.74 (3.63, 3.84)	3.52 (3.40, 3.64)	3.38	0.001
				
Physical environment	4.06 (3.99, 4.14)	3.85 (3.76, 3.94)	4.48	<0.001
Maintenance of equipment	4.05 (3.97, 4.14)	3.87 (3.77, 3.96)	3.50	0.001
Availability of food on call	2.82 (2.68, 2.96)	2.45 (3.30, 2.61)	4.16	<0.001
				
Clinical environment	4.00 (3.91, 4.09)	3.92 (3.82, 4.02)	1.36	0.18

* Items presented if statistically significant differences between non-procedural and procedural fellow reached p ≤ 0.001. All domains or items are presented in Additional file 1.

** Mean score scaled from 1 to 5, where 5 indicated very satisfied and 1 indicates very dissatisfied. Scores are adjusted to reflect a mean respondent by subspecialty grouping (procedure vs. non-procedural), computed for a PGY-4, and corrected for year of survey and facility nesting.

To measure the impact of domains on overall satisfaction, we measured the effect of domains on overall satisfaction score as the change in overall satisfaction (range 1 to 100) associated with each one-point change in the mean scale score for the domain (Likert scale with range 1 to 5). Each domain provided a statistically significant contribution to the overall score for both procedural and non-procedural trainees (data not shown). A comparison of the impact of each domain on overall satisfaction revealed differences between procedural and non-procedural fellows, with learning environment and personal experience contributing more to overall satisfaction for procedural fellows than for non-procedural fellows (Table 4). However, the rank order of the impact of each domain on overall satisfaction was similar, with both procedural and non-procedural trainees ranking personal experience the highest, followed by learning environment.

**Table 4:** Adjusted effects of each domain score on overall satisfaction for non-procedural and procedural fellows

	Non-procedural fellows	Procedural fellows		
Category	Mean (95% CI)*	Mean (95% CI)	*t*	*p*
Clinical faculty/preceptors	6.61 (5.81, 7.41)	7.86 (7.07, 8.65)	2.2	0.03
Personal experience	8.93 (8.06, 9.79)	11.34 (10.48, 12.20)	3.9	<0.001
Learning environment	7.83 (7.16, 8.50)	9.53 (8.81, 10.24)	3.4	0.001
Working environment	6.95 (6.24, 7.66)	7.60 (6.87, 8.34)	1.3	0.21
Physical environment	5.12 (4.32, 5.92)	5.98 (5.21, 6.74)	1.5	0.13
Clinical environment	7.24 (6.52, 7.95)	7.29 (6.56, 8.01)	0.1	0.92
				

*Effect size is the increase in overall satisfaction, measured on a 100-point scale, per increase in domain score, measured on a 5-point scale. Effect sizes were adjusted to reflect mean respondent by subspecialty grouping (procedural vs. non-procedural), computed for a PGY-4, and corrected for year of survey and facility nesting.

### Satisfaction with items within domains

Results for non-procedural fellows showed a consistent pattern of greater satisfaction on individual items, both in number of items and in magnitude (Additional file 1). Non-procedural fellows reported significantly higher satisfaction with: accessibility and availability of clinical faculty and preceptors; timeliness of feedback; fairness in evaluation; clinical faculty and preceptors' patient-oriented nature; time for learning; teaching conferences; morale of ancillary and support staff; laboratory services; ancillary and support staff; maintenance of equipment; availability of food on call; and continuity of relationship with patients (Additional file 1). Procedural fellows reported higher satisfaction with only two of 81 items (timely performance of necessary procedures/surgeries and degree of autonomy); these differences did not achieve statistical significance.

Within the domain of clinical faculty and preceptor, the rank order of satisfaction with items was similar, with highest satisfaction reported for approachability and openness, clinical skills, patient-oriented, and quality of faculty. For the domain of personal experience, the rank order of satisfaction with items was also similar, with highest satisfaction reported for relationship with patients, appreciation of respondent's work by patients, personal reward and personal responsibility for patient care.

We calculated the contribution of each item to its domain satisfaction score, measuring the change in the respective domain score associated with each one-point change on the Likert scale for individual items. Those items with larger effect sizes are listed in Table 5 and a more comprehensive listing is found in Additional file 2. For both types of trainees, most items within clinical faculty/preceptors were strongly associated with domain satisfaction, as were many items within the domain of personal experience. All items were found to have a statistically significant association with the respective domain score, both for procedural and non-procedural fellows (Additional file 2). Only a few items had statistically significant differences in impact on satisfaction for the two types of fellows (Additional file 2). Cleanliness of facilities and housekeeping, balance of personal and professional life, parking and level of job stress contributed more strongly to overall satisfaction for procedural fellows; however, these items contributed only modestly to the overall score and many were associated with lower overall domain satisfaction scores.

**Table 5:** Domain items with the largest* adjusted effect sizes on domain scores

	Non-procedural fellows	Procedural fellows		
Category	adj. effect (95% CI)	adj. effect (95% CI)	*t*	*p*
Clinical faculty/preceptors				
Clinical skills**	0.88 (0.84, 0.92)	0.88 (0.84, 0.93)	0.1	0.90
Teaching ability	0.84 (0.80, 0.87)	0.83 (0.79, 0.86)	0.4	0.67
Interest in teaching	0.76 (0.73, 0.80)	0.76 (0.73, 0.79)	0.1	0.92
Accessibility/availability	0.69 (0.64, 0.73)	0.71 (0.67, 0.75)	0.8	0.45
Approachability/openness	0.81 (0.76, 0.86)	0.78 (0.73, 0.82)	0.9	0.38
Fairness in evaluation	0.72 (0.68, 0.77)	0.74 (0.70, 0.78)	0.6	0.53
Role models	0.75 (0.72, 0.77)	0.75 (0.73, 0.78)	0.4	0.67
Patient-oriented	0.86 (0.82, 0.90)	0.80 (0.76, 0.84)	0.4	0.02
Quality of faculty	0.87 (0.83, 0.90)	0.83 (0.80, 0.87)	1.3	0.19
Evidence-based clinical practice	0.78 (0.74, 0.82)	0.84 (0.80, 0.88)	2.2	0.03
				
Learning environment				
Working with patients	0.71 (0.66, 0.76)	0.70 (0.65, 0.76)	0.1	0.90
Preparation for clinical practice	0.69 (0.65, 0.73)	0.72 (0.68, 0.76)	0.9	0.38
Preparation for future training	0.72 (0.68, 0.76)	0.75 (0.71, 0.79)	1.0	0.31
				
Clinical environment				
Ability to get the best care for patients	0.76 (0.73, 0.79)	0.76 (0.73, 0.79)	0.1	0.92
				
Physical environment				
Facility maintenance/upkeep	0.67 (0.63, 0.71)	0.70 (0.66, 0.74)	1.0	0.31
Lighting	0.73 (0.68, 0.78)	0.72 (0.67, 0.77)	0.3	0.79
Facility cleanliness/housekeeping	0.63 (0.59, 0.67)	0.73 (0.69, 0.77)	3.6	<0.001
				
Personal experience				
Relationship with patients	0.75 (0.68, 0.81)	0.75 (0.68, 0.81)	0.1	0.99
Enjoyment of respondent's work	0.73 (0.69, 0.77)	0.81 (0.77, 0.85)	2.9	0.004
Personal responsibility for patients	0.62 (0.57, 0.66)	0.71 (0.66, 0.76)	2.7	0.008
Enhancement of clinical knowledge/skills	0.78 (0.74, 0.81)	0.83 (0.79, 0.86)	2.0	0.05

*Items presented if the adjusted effect size for either non-procedural or procedural fellows was > = 0.70. Additional file 2 provides adjusted effect sizes for all LPS items.

**Effect size equals the change in the domain score per unit increase in the item score, adjusted to reflect a mean respondent by subspecialty grouping (procedural vs. non-procedural), computed for a PGY-4, and corrected for year of survey and facility nesting.

## Discussion

Our study is the first to comprehensively survey internal medicine subspecialty fellow satisfaction across multiple programs and compare perceptions between procedural and non-procedural fellows. Overall satisfaction with VA training is similar between procedural and non-procedural fellows in internal medicine, but differences exist at the item and domain-level.

The LPS is a validated survey instrument with robust psychometric properties and content and face validity that has been used to evaluate trainee satisfaction across a large, relatively uniform healthcare system. Previous studies have demonstrated its usefulness in comparing trainee perceptions across disciplines. Keitz et al found that the LPS functioned well in discriminating differences between different types of learners in VA [2]. Cannon et al extended the scope of the LPS by comparing satisfaction between medical students and residents [1]. Analysis of differences in subspecialty fellow satisfaction provides valuable information to GME leaders for program development and extends the utility of the LPS for evaluating satisfaction in graduate medical education.

There were similarities between our findings and those of Keitz et al [2] and Cannon et al [1] with respect to overall satisfaction and domain satisfaction. Overall satisfaction was similar for fellows, different types of residents [1,2] and medical students [1]. With the exception of physical environment, procedural and non-procedural fellows had similar domain satisfaction, as was seen with both medical students and residents [1]. Like medical students [1] and residents [1,2], fellows reported highest satisfaction with the domain of clinical faculty and preceptors. All three studies found that learning environment contributed highly to overall satisfaction, although in our study this was more so for procedural fellows. Unlike in the previous studies utilizing the LPS, our study examined the personal experience domain and found that personal experience was the domain most strongly associated with overall satisfaction for both procedural and non-procedural fellows, with both reporting similarly high levels of satisfaction with relationship with patients, appreciation of respondent's work by patients, personal reward and personal responsibility for patient care.

While domain satisfaction was similar across a wide spectrum of learners in all three studies, individual items within domains and their contribution to overall domain score provided more distinction between different types of learners. For example, procedural fellows reported lower satisfaction with several items in the clinical faculty/preceptor domain including accessibility/availability, timeliness of feedback, fairness of evaluation and patient orientation. Keitz et al found similar results for surgical residents who reported lower satisfaction with accessibility and availability of faculty and preceptors as compared to less procedural residents [2]. For subspecialty fellows, item differences were seen in equipment maintenance and food on-call, with procedural fellows expressing lower satisfaction. These results may reflect differing needs of procedural fellows who use diagnostic and therapeutic equipment more frequently and may be more likely to be on-call overnight in the hospital.

Within the working environment, non-procedural fellows reported higher satisfaction with ancillary/support staff morale, laboratory services, and ancillary/support staff. In a previous study, differences were also found between medical students and residents [1], and among residents, with internal medicine residents least satisfied with these items [2]. The relatively lower levels of satisfaction with these items among procedural fellows and internal medicine residents may reflect higher intensity of interactions with these services and therefore a different level of expectation compared to other types of learners. Whereas past studies suggested that such differences in satisfaction may be related to the differences in types of training programs [2], current results showing differences between procedural and non-procedural fellows may reflect degrees to which the same parts of the VA healthcare system intersect differently with the training goals of different program types and available training infrastructure.

Levels of satisfaction may reflect those attitudes and values that influenced physicians' choice of specialty training. A number of studies have evaluated predictors of subspecialty choice among residents and satisfaction with career choice among practicing physicians. In a study of factors associated with subspecialty choice of Canadian internal medicine residents, Horn et al found that residents pursuing non-procedural fellowships were more concerned with issues related to lifestyle, stress, work hours, leisure hours, and patient populations than those pursuing procedural fellowships [3]. Other studies have found that lifestyle [4-7], mentorship [3], faculty influence [4,5,8], role models [3,9,10], resident clinical experience [3-5,8] and high sense of satisfaction of fellows [8] are important factors in trainee selection of specialty training. Our study showed that personal experience (including lifestyle, stress and fatigue) and clinical faculty/preceptors (including mentoring and role models) contributed most significantly to overall satisfaction for both types of trainees, suggesting that improvements in these areas could lead to higher learner satisfaction and possibly more successful recruitment. Furthermore, differences in satisfaction with career choice have been noted between primary care and specialty residents [11] as well as procedure and non-procedure-based practicing physicians [12], suggesting that data on fellow satisfaction may provide useful information for residents in guiding career choices.

Quality in graduate medical education programs is complex and has many crucial components such as curriculum, trainee competency, and faculty development. Learner satisfaction with training is another crucial component for individual training programs [13], hospital systems [14], and national organizations [15]. With significant national focus on both changes in health care systems and regulatory requirements, particularly given forthcoming changes in Accreditation Council of Graduate Medical Education's Common Program Requirements, careful analysis of trainees' satisfaction with educational and work environments is essential to improving quality. Byrne et al advocated for the use of a comprehensive survey tool to examine residents' satisfaction with training, and demonstrated the use of such a tool to effect improvements within affiliated hospitals from one GME program [14]. The authors argued for the importance of a national, comprehensive survey tool to monitor trainee experience with the expressed goal of improving the training environment. In addition, monitoring the experience of trainees may provide advantages to individual programs in the form of longer accreditation cycle length [16]. Changes in trainee satisfaction should be monitored over time, and ideally both aggregate and facility-level data would be available to allow for analysis of variation between facilities.

Our study has several strengths. First, the LPS is a validated, comprehensive survey tool, providing key information regarding the work and learning environments in which the majority of physicians train. Second, the LPS targets learners within one large healthcare system, potentially limiting the degree to which the differences between healthcare systems in which trainees learn may affect the measured outcomes. Third, the LPS is designed to assess trainee perceptions across a full complement of subspecialty training programs, program training years and academic years. Finally, the results of this study are likely representative of fellow perceptions throughout the VA, since the distribution of respondents across subspecialties was nearly identical to the distribution of VA funded positions nationally for each subspecialty.

The study has several limitations. First, there were multiple comparisons performed in this study, which may have led to statistical error. For this reason, we set a threshold for statistical significance at p ≤ 0.001. Secondly, because the data are collected anonymously, we were unable to evaluate the changes in individual respondents over time, limiting the precision of our results. Thirdly, we were unable to determine the total number of fellows within the system and made the survey available but did not distribute the survey to them directly. Consequently, the estimated response rate was relatively low, and this strategy could result in selection bias. However, as mentioned, the distribution of fellow respondents mirrors the distribution of VA funding for each specialty. In addition, a comparison of common questions on the LPS and the American Academy of Medical Colleges (AAMC) medical student survey, which has a greater than 90% response rate, have demonstrated similar results suggesting that the sampling method for the LPS may not be subject to major selection bias. Finally, we collected the LPS data only for VA experiences, which may limit the applicability of the findings to other training settings. While most physicians train in a VA setting, further information about non-VA training settings would allow better understanding of how VA experience compares to the other sites where fellows train.

## Conclusions

VA internal medicine fellows of all subspecialties are generally satisfied with their VA training. For procedural and non-procedural fellows, satisfaction with the domains of clinical faculty and preceptors and personal experience contributed highly to the overall satisfaction score. Differences in satisfaction were evident in the comparison, and further study is needed to clarify whether the sources of these differences arise from dissimilarities in the learners themselves, the training needs of various disciplines or the parts of the healthcare system or infrastructure with which trainees interact. Better understanding of the factors associated with satisfaction among different types of fellows may assist residents in selecting training programs and may assist program directors and GME leaders in improving programs, thereby enhancing recruitment, improving fellow satisfaction with their training experience and enhancing educational outcomes.

## Competing interests

The authors declare that they have no competing interests.

## Authors' contributions

Authors CPK, SAK, TMK, DCA, JMB, BKC, GJH, and GWC all contributed to the design of the study, data collection, data collection assessment and evaluation, data analyses design and data analyses implementation, and drafting of the manuscript. Author CC contributed to data collection, data analysis and drafting of the manuscript. Author SCG contributed to data analysis and drafting of the manuscript. Author AW contributed to data analyses design and implementation, and drafting of the manuscript. All authors have read and provided approval to the manuscript as submitted.

## Pre-publication history

The pre-publication history for this paper can be accessed here:

http://www.biomedcentral.com/1472-6920/11/21/prepub

## Supplementary Material

Additional file 1 **Adjusted mean satisfaction scores for procedural and non-procedural fellows**. This table shows satisfaction with items within domains, comparing procedural and non-procedural fellows, and it includes all items examined in this study. Click here for file

Additional file 2 **Adjusted effect size of domain items on domain scores**. This table shows the adjusted effect sizes of all domain items on domain scores for procedural and non-procedural fellows and compares the effect size for both types of trainees. Click here for file
