# Supplementary material for: Training satisfaction for subspecialty fellows in internal medicine: Findings from the Veterans Affairs (VA) Learners' Perceptions Survey
Source: BMC Med Educ. 2011 May 17;11:21. doi: 10.1186/1472-6920-11-21 (PMC3121724; doi:10.1186/1472-6920-11-21)
Supplement: Additional file 1 — Adjusted mean satisfaction scores for procedural and non-procedural fellows. This table shows satisfaction with items within domains, comparing procedural and non-procedural fellows, and it includes all items examined in this study. [file 1472-6920-11-21-S1.PDF]

**Additional file 1 - Adjusted* mean satisfaction scores for procedural and non-procedural fellows**

| | Non-procedural fellows (95% CI) | Procedural fellows (95% CI) | *t* | *p* |
|---|---|---|---|---|
| Clinical faculty/preceptors | 4.42 (4.36, 4.48) | 4.32 (4.25, 4.40) | 2.41 | 0.016 |
| Clinical skills | 4.51 (4.46, 4.56) | 4.44 (4.37, 4.50) | 1.99 | 0.046 |
| Teaching ability | 4.44 (4.38, 4.50) | 4.33 (4.26, 4.41) | 2.69 | 0.007 |
| Interest in teaching | 4.45 (4.38, 4.51) | 4.33 (4.25, 4.41) | 2.81 | 0.005 |
| Research mentoring | 3.95 (3.86, 4.05) | 3.82 (3.71, 3.94) | 2.05 | 0.041 |
| Accessibility/availability | 4.48 (4.43, 4.54) | 4.35 (4.28, 4.42) | 3.35 | 0.001 |
| Approachability/openness | 4.59 (4.54, 4.65) | 4.50 (4.43, 4.56) | 2.46 | 0.014 |
| Timeliness of feedback | 4.33 (4.27, 4.40) | 4.19 (4.11, 4.27) | 3.30 | 0.001 |
| Fairness in evaluation | 4.46 (4.40, 4.52) | 4.31 (4.24, 4.38) | 3.66 | <0.001 |
| Role models | 4.34 (4.27, 4.41) | 4.20 (4.12, 4.29) | 2.95 | 0.003 |
| Mentoring by faculty | 4.31 (4.23, 4.39) | 4.21 (4.12, 4.31) | 1.85 | 0.064 |
| Patient-oriented | 4.50 (4.44, 4.56) | 4.37 (4.30, 4.44) | 3.29 | 0.001 |
| Quality of faculty | 4.48 (4.41, 4.55) | 4.36 (4.28, 4.44) | 2.88 | 0.004 |
| Evidence-based clinical practice | 4.43 (4.37, 4.50) | 4.36 (4.28, 4.43) | 1.85 | 0.065 |
| Learning environment | 4.17 (4.10, 4.24) | 4.11 (4.03, 4.19) | 1.35 | 0.18 |
| Working with patients | 4.43 (4.37, 4.50) | 4.36 (4.29, 4.43) | 1.79 | 0.073 |
| Degree of supervision | 4.40 (4.34, 4.46) | 4.37 (4.30, 4.45) | 0.61 | 0.54 |
| Degree of autonomy | 4.56 (4.51, 4.61) | 4.57 (4.51, 4.62) | 0.27 | 0.79 |
| Amount of non-education "scut" work | 3.80 (3.70, 3.90) | 3.62 (3.50, 3.74) | 2.69 | 0.007 |
| Interdisciplinary approach | 4.16 (4.09, 4.23) | 4.02 (3.93, 4.10) | 2.94 | 0.003 |
| Preparation for clinical practice | 4.21 (4.14, 4.27) | 4.12 (4.03, 4.20) | 2.12 | 0.035 |
| Preparation for future training | 4.22 (4.15, 4.29) | 4.18 (4.10, 4.26) | 0.98 | 0.33 |
| Preparation for business aspects | 3.17 (3.06, 3.27) | 3.12 (3.00, 3.25) | 0.59 | 0.56 |
| Time for learning | 4.19 (4.11, 4.26) | 3.99 (3.90, 4.08) | 4.05 | <0.001 |
| Access to specialty expertise | 4.18 (4.10, 4.25) | 4.11 (4.03, 4.20) | 1.35 | 0.18 |
| Teaching conferences | 4.02 (3.94, 4.10) | 3.84 (3.75, 3.94) | 3.23 | 0.001 |
| Quality of care | 4.12 (4.04, 4.21) | 4.07 (3.97, 4.17) | 0.95 | 0.34 |
| Culture of patient safety | 4.14 (4.06, 4.22) | 4.09 (3.99, 4.18) | 0.92 | 0.36 |
| Spectrum of patient problems | 4.26 (4.20, 4.32) | 4.21 (4.14, 4.29) | 1.18 | 0.24 |
| Diversity of patients | 4.00 (3.92, 4.07) | 3.90 (3.81, 4.00) | 1.76 | 0.078 |

| | | | | |
|---|---|---|---|---|
| Clinical environment | 4.00 (3.91, 4.09) | 3.92 (3.82, 4.02) | 1.36 | 0.18 |
| Hours at work | 4.43 (4.38, 4.49) | 4.36 (4.28, 4.43) | 2.04 | 0.042 |
| Number of inpatients admitted | 4.38 (4.32, 4.47) | 4.35 (4.27, 4.43) | 0.80 | 0.43 |
| Number of outpatients seen | 4.24 (4.16, 4.32) | 4.23 (4.13, 4.32) | 0.29 | 0.78 |
| Timely availability of outpatient appointments | 3.88 (3.78, 3.99) | 3.87 (3.74, 3.99) | 0.24 | 0.81 |
| Timely performance of procedures & surgery | 3.55 (3.43, 3.66) | 3.72 (3.58, 3.85) | 2.40 | 0.017 |
| Admitting patients in a timely fashion | 4.10 (4.01, 4.19) | 4.07 (3.97, 4.17) | 0.41 | 0.68 |
| Ability to use emerging therapies | 3.68 (3.58, 3.79) | 3.59 (3.46, 3.71) | 1.39 | 0.17 |
| How well physicians/nurses work together | 4.01 (3.92, 4.10) | 3.95 (3.84, 4.05) | 1.09 | 0.27 |
| How well physicians/ancillary staff work together | 3.92 (3.82, 4.02) | 3.86 (3.75, 3.98) | 0.74 | 0.46 |
| Getting test done timely fashion, weekdays | 3.91 (3.81, 4.02) | 3.84 (3.72, 3.96) | 1.01 | 0.32 |
| Timely testing, nights & weekends | 3.12 (2.98, 3.25) | 2.97 (2.82, 3.12) | 1.71 | 0.088 |
| Ease of getting patient records | 4.45 (4.38, 4.52) | 4.37 (4.28, 4.45) | 1.67 | 0.096 |
| Backup system of electronic medical records | 3.88 (3.78, 3.98) | 3.80 (3.68, 3.92) | 1.35 | 0.18 |
| Amount of paper work | 3.83 (3.73, 3.93) | 3.66 (3.54, 3.78) | 2.64 | 0.008 |
| Ability to get the best care for patients | 3.93 (3.84, 4.03) | 3.84 (3.73, 3.96) | 1.40 | 0.16 |
| Working environment | 4.12 (4.04, 4.19) | 4.00 (3.91, 4.08) | 2.40 | 0.017 |
| Faculty/preceptor morale | 4.34 (4.27, 4.41) | 4.24 (4.15, 4.33) | 2.16 | 0.031 |
| Ancillary/support staff morale | 3.84 (3.74, 3.94) | 3.54 (3.42, 3.66) | 4.59 | <0.001 |
| Peer group morale | 4.17 (4.10, 4.25) | 4.06 (3.98, 4.15) | 2.28 | 0.023 |
| Laboratory services | 3.99 (3.91, 4.07) | 3.81 (3.71, 3.91) | 3.38 | 0.001 |
| Radiology services | 3.76 (3.66, 3.86) | 3.67 (3.55, 3.78) | 1.32 | 0.19 |
| Ancillary/support staff | 3.74 (3.63, 3.84) | 3.52 (3.40, 3.64) | 3.38 | 0.001 |
| Call schedule | 4.36 (4.30, 4.43) | 4.30 (4.23, 4.38) | 1.24 | 0.22 |
| Computerized Patient Record System | 4.52 (4.46, 4.58) | 4.45 (4.37, 4.52) | 1.58 | 0.11 |
| Orientation program | 4.02 (3.94, 4.10) | 3.98 (3.88, 4.07) | 0.75 | 0.45 |
| Library services | 3.98 (3.89, 4.07) | 3.84 (3.73, 3.95) | 2.28 | 0.023 |
| Computer access | 4.36 (4.29, 4.43) | 4.27 (4.18, 4.35) | 2.02 | 0.044 |
| Internet access | 4.22 (4.14, 4.30) | 4.07 (3.97, 4.17) | 2.81 | 0.005 |
| Workspace | 4.19 (4.11, 4.27) | 4.05 (3.96, 4.14) | 2.79 | 0.005 |
| Physical environment | 4.06 (3.99, 4.14) | 3.85 (3.76, 3.94) | 4.48 | <0.001 |
| Convenience of facility location | 4.37 (4.30, 4.45) | 4.30 (4.22, 4.39) | 1.74 | 0.083 |
| Parking | 3.33 (3.21, 3.45) | 3.31 (3.17, 3.46) | 0.12 | 0.90 |

| | | | | |
|---|---|---|---|---|
| Personal safety | 4.34 (4.28, 4.41) | 4.30 (4.22, 4.38) | 1.15 | 0.25 |
| Availability of phones | 4.25 (4.18, 4.31) | 4.18 (4.09. 4.26) | 1.57 | 0.12 |
| Availability of needed equipment | 4.04 (3.96, 4.12) | 3.87 (3.77, 3.97) | 3.15 | 0.002 |
| Maintenance of equipment | 4.05 (3.97, 4.14) | 3.87 (3.77, 3.96) | 3.50 | 0.001 |
| Facility maintenance/upkeep | 4.14 (4.06, 4.22) | 4.00 (3.91, 4.10) | 2.75 | 0.006 |
| Lighting | 4.31 (4.25, 4.38) | 4.21 (4.13, 4.29) | 2.51 | 0.012 |
| Heating/air conditioning | 4.21 (4.14, 4.29) | 4.12 (4.04, 4.23) | 1.69 | 0.092 |
| Facility cleanliness/housekeeping | 4.14 (4.06, 4.22) | 4.07 (3.97, 4.16) | 1.58 | 0.12 |
| Call rooms | 3.85 (3.72, 3.98) | 3.65 (3.50, 3.80) | 2.39 | 0.017 |
| Availability of food on call | 2.82 (2.68, 2.96) | 2.45 (3.30, 2.61) | 4.16 | <0.001 |
| Personal experience | 4.35 (4.29, 4.41) | 4.24 (4.17, 4.31) | 2.83 | 0.005 |
| Personal support | 4.48 (4.43, 4.54) | 4.45 (4.38, 4.52) | 0.96 | 0.34 |
| Personal reward | 4.34 (4.28, 4.40) | 4.32 (4.24, 4.39) | 0.52 | 0.60 |
| Relationship with patients | 4.58 (4.53, 4.63) | 4.53 (4.46, 4.59) | 1.59 | 0.11 |
| Appreciation of respondent's work by faculty | 4.36 (4.28, 4.44) | 4.27 (4.17, 4.36) | 1.89 | 0.061 |
| Appreciation of respondent's work by patients | 4.51 (4.46, 4.57) | 4.48 (4.41, 4.54) | 0.97 | 0.33 |
| Balance of personal and professional life | 4.29 (4.22, 4.35) | 4.19 (4.11, 4.27) | 2.16 | 0.031 |
| Enjoyment of respondent's work | 4.38 (4.32, 4.44) | 4.32 (4.24, 4.40) | 1.42 | 0.16 |
| Level of job stress | 4.13 (4.06, 4.20) | 4.05 (3.96, 4.14) | 1.69 | 0.091 |
| Level of fatigue | 4.23 (4.15, 4.31) | 4.13 (4.03, 4.22) | 1.87 | 0.062 |
| Continuity of relationship with patients | 4.25 (4.17, 4.33) | 4.07 (3.98, 4.17) | 3.38 | 0.001 |
| Personal responsibility for patient care | 4.45 (4.39, 4.52) | 4.39 (4.31, 4.47) | 1.32 | 0.19 |
| Quality of care respondent’s patients receive | 4.24 (4.16, 4.32) | 4.15 (4.06, 4.24) | 1.83 | 0.067 |
| Enhancement of clinical knowledge/skills | 4.38 (4.32, 4.44) | 4.29 (4.21, 4.37) | 2.10 | 0.036 |

*Ajusted to reflect mean respondent by subspecialty grouping (procedural vs. non-procedural), computed for a PGY-4, and corrected for year of survey and facility nesting. Scale ranges from 1 to 5, where 5 reflects “very satisfied.”
