# Supplementary material for: Training satisfaction for subspecialty fellows in internal medicine: Findings from the Veterans Affairs (VA) Learners' Perceptions Survey
Source: BMC Med Educ. 2011 May 17;11:21. doi: 10.1186/1472-6920-11-21 (PMC3121724; doi:10.1186/1472-6920-11-21)
Supplement: Additional file 2 — Adjusted effect size of domain items on domain scores. This table shows the adjusted effect sizes of all domain items on domain scores for procedural and non-procedural fellows and compares the effect size for both types of trainees. [file 1472-6920-11-21-S2.PDF]

Additional file 2 - Adjusted effect size of domain items on domain scores

	Non-procedural fellows			Procedural fellows			Effect Size Difference		
	adj. effect (95% CI)*	*t*	*p*	adj. effect (95% CI)	*t*	*p*	Mean	*t*	*p*
Clinical faculty/preceptors									
Clinical skills	0.88 (0.84, 0.92)	39.0	<0.001	0.88 (0.84, 0.93)	42.1	<0.001	0.00 (-0.06, 0.06)	0.1	0.90
Teaching ability	0.84 (0.80, 0.87)	46.6	<0.001	0.83 (0.79, 0.86)	49.7	<0.001	-0.01 (-0.06, 0.04)	-0.4	0.67
Interest in teaching	0.76 (0.73, 0.80)	42.4	<0.001	0.76 (0.73, 0.79)	44.5	<0.001	0.00 (-0.05, 0.05)	-0.1	0.92
Research mentoring	0.43 (0.39, 0.47)	19.9	<0.001	0.49 (0.45, 0.53)	23.8	<0.001	0.06 (0.00, 0.12)	2.0	0.05
Accessibility/availability	0.69 (0.64, 0.73)	29.5	<0.001	0.71 (0.67, 0.75)	33.3	<0.001	0.02 (-0.04, 0.09)	0.8	0.45
Approachability/openness	0.81 (0.76, 0.86)	32.5	<0.001	0.78 (0.73, 0.82)	35.4	<0.001	-0.03 (-0.09, 0.04)	-0.9	0.38
Timeliness of feedback	0.62 (0.58, 0.66)	30.1	<0.001	0.66 (0.62, 0.70)	33.5	<0.001	0.04 (-0.16, 0.10)	1.4	0.16
Fairness in evaluation	0.72 (0.68, 0.77)	32.4	<0.001	0.74 (0.70, 0.78)	33.9	<0.001	0.02 (-0.04, 0.08)	0.6	0.53
Role models	0.75 (0.72, 0.77)	50.3	<0.001	0.75 (0.73, 0.78)	52.9	<0.001	0.01 (-0.03, 0.05)	0.4	0.67
Mentoring by faculty	0.68 (0.64, 0.71)	39.6	<0.001	0.70 (0.67, 0.73)	43.5	<0.001	0.02 (-0.02, 0.07)	1.0	0.30
Patient-oriented	0.86 (0.82, 0.90)	42.2	<0.001	0.80 (0.76, 0.84)	44.2	<0.001	-0.06 (-0.12, -0.01)	-2.3	0.02
Quality of faculty	0.87 (0.83, 0.90)	47.3	<0.001	0.83 (0.80, 0.87)	52.6	<0.001	-0.03 (-0.08, 0.02)	-1.3	0.19
Evidence-based clinical practice	0.78 (0.74, 0.82)	38.3	<0.001	0.84 (0.80, 0.88)	41.7	<0.001	0.06 (0.01, 0.12)	2.2	0.03
Learning environment									
Working with patients	0.71 (0.66, 0.76)	26.0	<0.001	0.70 (0.65, 0.76)	24.9	<0.001	-0.01 (-0.08, 0.07)	-0.1	0.90
Degree of supervision	0.62 (0.57, 0.67)	25.0	<0.001	0.67 (0.62, 0.72)	25.2	<0.001	0.05 (-0.02, 0.12)	1.4	0.17
Degree of autonomy	0.65 (0.59, 0.72)	19.1	<0.001	0.70 (0.63, 0.77)	19.3	<0.001	0.04 (-0.06, 0.14)	0.9	0.40
Amount of non-education "scut" work	0.42 (0.38, 0.45)	23.5	<0.001	0.42 (0.40, 0.46)	23.7	<0.001	0.01 (-0.04, 0.06)	0.2	0.83
Interdisciplinary approach	0.59 (0.55, 0.63)	28.2	<0.001	0.65 (0.61, 0.70)	30.0	<0.001	0.07 (0.01, 0.12)	2.2	0.03
Preparation for clinical practice	0.69 (0.65, 0.73)	34.0	<0.001	0.72 (0.68, 0.76)	34.2	<0.001	0.03 (-0.03, 0.83)	0.9	0.38
Preparation for future training	0.72 (0.68, 0.76)	35.4	<0.001	0.75 (0.71, 0.79)	35.2	<0.001	0.03 (-0.28, 0.09)	1.0	0.31
Preparation for business aspects	0.37 (0.33, 0.41)	20.0	<0.001	0.39 (0.35, 0.43)	20.7	<0.001	0.02 (-0.03, 0.07)	0.8	0.45
Time for learning	0.60 (0.56, 0.65)	27.7	<0.001	0.56 (0.52, 0.61)	26.2	<0.001	-0.04 (-0.10, 0.02)	-1.3	0.19
Access to specialty expertise	0.57 (0.53, 0.61)	26.5	<0.001	0.65 (0.61, 0.70)	29.8	<0.001	0.82 (0.22, 0.14)	2.7	0.007

Teaching conferences	0.54 (0.50, 0.58)	24.6	<0.001	0.53 (0.49, 0.57)	25.8	<0.001	-0.01 (0.07, 0.05)	-0.2	0.81
Quality of care	0.69 (0.66, 0.73)	39.4	<0.001	0.68 (0.64, 0.71)	37.2	<0.001	-0.01 (-0.06, 0.04)	-0.5	0.64
Culture of patient safety	0.61 (0.57, 0.65)	31.5	<0.001	0.63 (0.59, 0.67)	31.3	<0.001	0.02 (-0.03, 0.08)	0.8	0.43
Spectrum of patient problems	0.60 (0.56, 0.65)	24.2	<0.001	0.64 (0.59, 0.69)	24.2	<0.001	0.04 (-0.03, 0.01)	1.0	0.32
Diversity of patients	0.48 (0.43, 0.53)	19.1	<0.001	0.52 (0.47, 0.56)	21.3	<0.001	0.04 (-0.03, 0.11)	1.1	0.27
Clinical environment									
Hours at work	0.63 (0.57, 0.70)	18.1	<0.001	0.64 (0.56, 0.71)	17.1	<0.001	0.00 (-0.97, 0.10)	0.1	0.96
Number of inpatients admitted	0.68 (0.60, 0.76)	16.6	<0.001	0.64 (0.56, 0.72)	16.1	<0.001	-0.04 (-0.15, 0.07)	-0.7	0.49
Number of outpatients seen	0.54 (0.48, 0.59)	19.2	<0.001	0.56 (0.50, 0.62)	17.7	<0.001	0.03 (-0.06, 0.11)	0.6	0.55
Timely availability of outpatient appointments	0.49 (0.45, 0.54)	22.5	<0.001	0.48 (0.44, 0.53)	20.2	<0.001	-0.01 (-0.08, 0.05)	-0.4	0.70
Timely performance procedures & surgery	0.50 (0.46, 0.54)	26.6	<0.001	0.56 (0.52, 0.60)	28.2	<0.001	0.06 (0.00, 0.11)	2.1	0.04
Admitting patients in a timely fashion	0.65 (0.59, 0.70)	23.4	<0.001	0.67 (0.61, 0.72)	24.8	<0.001	0.02 (-0.06, 0.09)	0.4	0.66
Ability to use emerging therapies	0.46 (0.42, 0.50)	22.6	<0.001	0.50 (0.46, 0.55)	23.4	<0.001	0.05 (-0.01, 0.10)	1.6	0.12
How well physicians/nurses work together	0.62 (0.57, 0.66)	28.1	<0.001	0.66 (0.62, 0.71)	28.8	<0.001	0.05 (-0.01, 0.11)	1.5	0.13
How well physicians/ancillary staff work together	0.62 (0.58, 0.66)	30.2	<0.001	0.64 (0.60, 0.68)	30.4	<0.001	0.02 (-0.04, 0.08)	0.7	0.47
Getting test done timely fashion, weekdays	0.62 (0.58, 0.65)	31.3	<0.001	0.63 (0.59, 0.67)	30.6	<0.001	0.01 (-0.04, 0.07)	0.4	0.65
Timely testing, nights/ weekends	0.47 (0.44, 0.51)	25.9	<0.001	0.47 (0.44, 0.51)	25.7	<0.001	0.00 (-.0.05, 0.05)	-0.1	0.96
Ease of getting patient records	0.52 (0.45, 0.58)	15.3	<0.001	0.47 (0.41, 0.54)	14.3	<0.001	-0.04 (-0.14, 0.05)	-0.9	0.35
Backup system of electronic medical records	0.45 (0.40, 0.50)	18.1	<0.001	0.46 (0.41, 0.51)	17.9	<0.001	0.01 (-0.06, 0.08)	0.3	0.76
Amount of paper work	0.48 (0.44, 0.53)	22.5	<0.001	0.49 (0.45, 0.53)	22.7	<0.001	0.01 (-0.05, 0.07)	0.2	0.84
Ability to get best care for patients	0.76 (0.73, 0.79)	49.1	<0.001	0.76 (0.73, 0.79)	49.7	<0.001	0.00 (-0.04, 0.04)	0.1	0.92
Working environment									
Faculty/preceptor morale	0.54 (0.50, 0.59)	22.5	<0.001	0.55 (0.50, 0.59)	22.8	<0.001	0.00 (-0.06, 0.69)	0.1	0.93
Ancillary/support staff morale	0.49 (0.45, 0.52)	28.3	<0.001	0.50 (0.46, 0.53)	28.5	<0.001	0.01 (-0.04, 0.06)	0.5	0.63
Peer group morale	0.62 (0.57, 0.66)	26.8	<0.001	0.60 (0.55, 0.64)	26.3	<0.001	-0.02 (-0.08, 0.04)	-0.6	0.53
Laboratory services	0.53 (0.49, 0.57)	25.4	<0.001	0.56 (0.52, 0.60)	25.7	<0.001	0.03 (-0.03, 0.09)	0.9	0.38
Radiology services	0.45 (0.41, 0.49)	24.5	<0.001	0.51 (0.47, 0.55)	25.7	<0.001	0.06 (0.00, 0.11)	2.1	0.03
Ancillary/support staff	0.47 (0.44, 0.51)	27.7	<0.001	0.54 (0.50, 0.57)	30.5	<0.001	0.06 (0.01, 0.11)	2.5	0.01
Call schedule	0.57 (0.51, 0.63)	18.4	<0.001	0.65 (0.58, 0.71)	20.7	<0.001	0.08 (-0.01, 0.16)	1.8	0.07
Computerized Patient Record System	0.56 (0.50, 0.62)	18.5	<0.001	0.49 (0.43, 0.55)	16.2	<0.001	-0.08 (-0.16, 0.01)	-1.8	0.07
Orientation program	0.47 (0.43, 0.52)	21.9	<0.001	0.54 (0.49, 0.59)	22.1	<0.001	0.07 (0.00, 0.13)	2.0	0.05

Library services	0.44 (0.39, 0.49)	18.9	<0.001	0.48 (0.43, 0.53)	19.8	<0.001	0.04 (-0.03, 0.10)	1.2	0.25
Computer access	0.53 (0.48, 0.58)	20.7	<0.001	0.51 (0.46, 0.56)	19.8	<0.001	-0.02 (-0.09, 0.05)	-0.5	0.64
Internet access	0.46 (0.42, 0.51)	20.6	<0.001	0.45 (0.41, 0.50)	20.5	<0.001	-0.01 (-0.07, 0.05)	-0.3	0.75
Workspace	0.55 (0.51, 0.59)	25.6	<0.001	0.57 (0.52, 0.61)	25.8	<0.001	0.02 (-0.04, 0.08)	0.6	0.58
Physical environment									
Convenience of facility location	0.48 (0.42, 0.53)	16.8	<0.001	0.57 (0.51, 0.62)	19.4	<0.001	0.09 (0.01, 0.17)	2.2	0.03
Parking	0.21 (0.17, 0.25)	11.3	<0.001	0.30 (0.26, 0.34)	15.3	<0.001	0.09 (0.39, 0.01)	3.4	0.001
Personal safety	0.60 (0.54, 0.65)	21.5	<0.001	0.61 (0.55, 0.66)	23.1	<0.001	0.01 (-0.07, 0.08)	0.2	0.88
Availability of phones	0.50 (0.46, 0.55)	20.8	<0.001	0.56 (0.51, 0.61)	22.4	<0.001	0.06 (-0.01, 0.13)	1.7	0.94
Availability of needed equipment	0.58 (0.54, 0.62)	28.9	<0.001	0.65 (0.61, 0.69)	32.4	<0.001	0.06 (0.01, 0.12)	2.3	0.02
Maintenance of equipment	0.62 (0.57, 0.66)	29.6	<0.001	0.62 (0.58, 0.66)	31.1	<0.001	-0.01 (-0.05, 0.65)	0.3	0.75
Facility maintenance/upkeep	0.67 (0.63, 0.71)	31.8	<0.001	0.70 (0.66, 0.74)	33.7	<0.001	0.03 (-0.03, 0.09)	1.0	0.31
Lighting	0.73 (0.68, 0.78)	28.9	<0.001	0.72 (0.67, 0.77)	27.6	<0.001	-0.01 (-0.08, 0.06)	-0.3	0.79
Heating/air conditioning	0.53 (0.48, 0.57)	23.2	<0.001	0.60 (0.55, 0.65)	25.4	<0.001	0.07 (0.01, 0.13)	2.2	0.03
Facility cleanliness/housekeeping	0.63 (0.59, 0.67)	32.1	<0.001	0.73 (0.69, 0.77)	35.3	<0.001	0.10 (0.47, 0.16)	3.6	<0.001
Call rooms	0.53 (0.47, 0.59)	17.2	<0.001	0.57 (0.52, 0.62)	22.8	<0.001	0.05 (-0.32, 0.12)	1.2	0.25
Availability of food on call	0.33 (0.28, 0.37)	14.7	<0.001	0.37 (0.32, 0.41)	16.8	<0.001	0.04 (-0.22, 0.10)	1.3	0.21
Personal experience									
Personal support	0.61 (0.55, 0.67)	20.8	<0.001	0.68 (0.62, 0.73)	23.1	<0.001	0.06 (-0.02, 0.15)	1.6	0.12
Personal reward	0.64 (0.60, 0.69)	29.4	<0.001	0.70 (0.66, 0.74)	30.9	<0.001	0.06 (-0.01, 0.12)	1.8	0.07
Relationship with patients	0.75 (0.68, 0.81)	23.5	<0.001	0.75 (0.68, 0.81)	23.7	<0.001	0.00 (-0.09, 0.09)	-0.1	0.99
Appreciation of respondent's work by faculty	0.51 (0.47, 0.55)	23.2	<0.001	0.52 (0.48, 0.56)	25.1	<0.001	0.01 (-0.05, 0.07)	0.4	0.71
Appreciation of respondent's work by patients	0.67 (0.61, 0.72)	23.1	<0.001	0.66 (0.60, 0.72)	22.0	<0.001	0.00 (-0.08, 0.08)	0.0	0.97
Balance of personal and professional life	0.55 (0.51, 0.59)	25.0	<0.001	0.67 (0.62, 0.71)	27.7	<0.001	0.12 (0.05, 0.18)	3.3	<0.001
Enjoyment of respondent's work	0.73 (0.69, 0.77)	38.5	<0.001	0.81 (0.77, 0.85)	40.8	<0.001	0.08 (0.25, 0.13)	2.9	0.004
Level of job stress	0.54 (0.50, 0.58)	27.4	<0.001	0.64 (0.60, 0.68)	30.4	<0.001	0.10 (0.04, 0.16)	3.5	0.001
Level of fatigue	0.52 (0.48, 0.57)	22.8	<0.001	0.54 (0.50, 0.59)	23.4	<0.001	0.02 (-0.04, 0.08)	0.6	0.53
Continuity of relationship with patients	0.50 (0.46, 0.55)	23.9	<0.001	0.54 (0.50, 0.58)	26.0	<0.001	0.03 (-0.03, 0.89)	1.1	0.28
Personal responsibility for patient care	0.62 (0.57, 0.66)	26.5	<0.001	0.71 (0.66, 0.76)	28.3	<0.001	0.09 (0.02, 0.05)	2.7	0.008
Quality of care respondent's patients receive	0.60 (0.56, 0.64)	30.8	<0.001	0.64 (0.60, 0.68)	33.5	<0.001	0.04 (-0.01, 0.96)	1.6	0.12
Enhancement of clinical knowledge & skills	0.78 (0.74, 0.81)	42.0	<0.001	0.83 (0.79, 0.86)	45.4	<0.001	0.05 (0.00, 0.10)	2.0	0.05

*Effect size equals the change in the domain score per unit increase in the item score, adjusted to reflect a mean respondent by subspecialty grouping (procedural vs. non-procedural), computed for a PGY-4, and corrected for year of survey and facility nesting.
